# Conflicting intentions: rectifying the consistency requirements

**DOI:** 10.1007/s11098-018-1049-z

**Published:** 2018-02-20

**Authors:** Hein Duijf, Jan Broersen, John-Jules Ch. Meyer

**Affiliations:** 1 Theoretical Philosophy group, Department of Philosophy and Religious Studies, Utrecht University, 3508 TB Utrecht, The Netherlands; 2 Intelligent Systems group, Department of Information and Computing Sciences, Utrecht University, P.O. Box 80.089, 3508 TB Utrecht, The Netherlands

**Keywords:** Intention, Consistency requirements, Norms of rationality, Bratman

## Abstract

Many philosophers are convinced that rationality dictates that one’s overall set of intentions be consistent. The starting point and inspiration for our study is Bratman’s planning theory of intentions. According to this theory, one needs to appeal to the fulfilment of characteristic planning roles to justify norms that apply to our intentions. Our main objective is to demonstrate that one can be rational despite having mutually inconsistent intentions. Conversely, it is also shown that one can be irrational despite having a consistent overall set of intentions. To overcome this paradox, we argue that it is essential for a successful planning system that one’s intentions are *practically consistent* rather than being consistent or applying an aggregation procedure. Our arguments suggest that a new type of norm is needed: whereas the consistency requirement focuses on rendering the contents of one’s intentions consistent, our new practical consistency requirement demands that one’s intentions be able to simultaneously and unconditionally guide one’s action. We observe that for intentions that conform to the ‘own-action condition’, the practical consistency requirement is equivalent to the traditional consistency requirement. This implies that the consistency requirement only needs to be amended in scenarios of choice under uncertainty.

## Introduction

We focus on two questions: why should our intentions respect certain norms of rationality?^1^ And which norms of rationality should our intentions respect? Michael Bratman’s (1987) influential planning theory of intentions is the starting point and inspiration for our enquiry. The consistency requirement, which says that an agent’s intentions need to be co-realizable, plays an important role in his theory. Our main objective is to argue that it can be rational to have mutually *in*consistent intentions.^2^ Moreover, we show that one can be irrational despite having mutually consistent intentions. Demands of rationality are thus independent of the consistency requirement.

Why should our intentions respect the consistency requirement? Note that our desires need not meet the consistency requirement: I can remain perfectly rational even though my desires conflict. We agree with Bratman (1987, p. 16) that our intentions, in contrast to our desires, are “conduct controlling pro-attitudes” that impose an unconditional commitment. These features are central to how our intentions guide and control our thought and action. We should arguably respect the consistency requirement *because* it is essential for the successful operation of this system of coordinated control. The rationality of an agent’s intentions thus derives from the operation of her planning system. We introduce an acceptance criterion that captures this argument: whenever conformity to a certain norm facilitates successful operation, this norm is to be accepted.

Should our intentions conform to the consistency requirement on this count? To address this question, a clear formulation of the consistency requirement is needed. We propose three alternative readings of Bratman’s (2009b) consistency requirement and dispute the claim that conformity to any of these consistency requirements is essential for a successful planning system. The three consistency requirements are neither too weak nor too strong; instead, they miss the mark on a more fundamental count: they fail to facilitate the successful guidance of our actions. This proves our main claim: one can be rational despite having mutually inconsistent intentions.

So which norms should our intentions respect? We argue that a different type of norm is required. To see this, we start by reconsidering the characteristic commitment of our intentions. Bratman (1987, p. 33) writes that an agent’s intentions provide a “filter of admissibility” for the available options. Because each intention induces an unconditional commitment, every one of the agent’s intentions provides such a filter of admissibility for her available options.^3^ We submit that an agent’s body of intentions *facilitates successful operation* when those intentions are able to simultaneously and unconditionally guide her actions, that is, when there is an action available that survives the filtering induced by each of her intentions.

Are scenarios of choice under *un*certainty essential to our argument for rectifying the consistency requirements? In the final part of this paper, we focus on what Bratman (2014, Sections 1.3 and 3.1) calls the “the own-action condition”. This condition roughly states that the content of each intention is composed of actions that are attributable to the agent herself. We show that the practical consistency requirement and the overall consistency requirement are equivalent for intentions that conform to this own-action condition. This prompts two insights: first, the practical consistency norm only differs from the overall consistency requirement for intentions that violate the own-action condition. This emphasizes that the practical consistency requirement is a natural extension of the overall consistency requirement to more general intentions. Second, the consistency requirements are only inessential for a successful planning system in scenarios of choice under *uncertainty*. In other words, only in scenarios of choice under uncertainty do we need to amend the widely accepted position that one’s intentions should be overall consistent.

The paper is set out as follows. To prove our main claims, we present possible worlds semantics for agency, possibility, and intentions in Sects. 2 and 3. This formal framework is used to propose three alternative formalizations of Bratman’s (2009b) consistency requirement: mutual consistency, overall consistency, and agglomeration. In Sect. 4, we trace the justification for the consistency requirements to a deeper norm of successful operation and state an acceptance criterion that echoes this justification. To specify this acceptance criterion in a formal way, we use a technical decision principle called the “sure-thing” principle in Sect. 5. This yields the norm of practical consistency, which states that an agent’s body of intentions should be composed of intentions such that there is an action available to her that is *optimal* with respect to every single one of her intentions. In Sect. 6 we prove our previously stated central results. We briefly reflect on the endorsed technical decision principle and trace our central results to two intuitive properties in Sect. 6.3. In Sect. 7 we show that the practical consistency norm is equivalent to the overall consistency norm when we restrict our enquiry to intentions that conform to the own-action condition. We end with a brief discussion.

## Agency

Our study of norms of rationality that apply to intentions is cast against the background of the theory of ‘seeing to it that’, abbreviated to STIT (Belnap et al. 2001; Horty 2001). Our STIT models are derived from the well-known framework of agents and choices in branching time developed by Belnap et al. (2001). For simplicity’s sake, we do not adopt these branching time models and instead use a standard possible worlds approach to model agency, possibility, and intentions at a single moment in time. A STIT model involves a set of possible worlds *W* and a set of available actions ${{\mathsf {Act}}}$.^4^ We may take the possible worlds to represent the possibilities that are still open, which neatly models the idea of indeterminism.^5^ Conversely, possible worlds outside *W* are no longer possible, or accessible. Given that the worlds in *W* are still open, an agent’s action (or choice) is viewed as restricting the possible worlds to a subset *K* of *W*. When an agent closes the door, the nature of her action is to constrain the possible worlds to those where the door is closed. Or, conversely, the nature of her action is to exclude the possible worlds in which the door is open. Hence, an action is identified with a subset *K* of the set of possible worlds *W*; the possible worlds outside of *K* are excluded by performing that action. This gives rise to the reading that an agent sees to it that $\varphi$ only if she performs an action *K*, thereby constraining the possible worlds to $\varphi$-worlds.

### **Definition 1**

*(STIT Model)* A (single-agent) STIT model is an ordered pair $\langle W,{\mathsf {Act}}\rangle$ consisting of a non-empty set of possible worlds *W* and a set of available actions ${\mathsf {Act}}\subseteq 2^W$, where ${\mathsf {Act}}$ is a partitioning of *W*.

## Intentions

Though philosophers have studied the various guises of intention, we restrict our attention to future-directed intentions as studied in the planning theory of intentions advanced by Bratman (1987).^6^ Bratman views future-directed intentions as plan states that have certain characteristic features. The following two features are most important for our current purposes: partiality and control of conduct.^7^

First, plans are typically “partial” (Bratman 1987, p. 2). Our intentions need not specify every detail of what we are doing now or in the future. My intention to buy groceries, for instance, need not specify every single step I will take on my way to the shop. Such total plans would obviously go beyond our cognitive capacities. There are two different types of future-directed intentions: I can intend to perform a certain action, or I can intend to realize a certain state of affairs. This difference may best be viewed as the distinction between action intentions and goal intentions.^8^ We focus primarily on intentions to realize a certain state of affairs, i.e. goal intentions. Therefore, we characterize an intention by a proposition, that is, by a set of possible worlds. Intuitively, an intention $J(\subseteq W)$ is an intention to realize exactly those aspects that all elements of *J* have in common. This neatly models the characteristic partiality of our intentions. The body of intentions of the agent is modelled by a collection ${\mathsf {Int}}$ of intentions. This gives rise to the reading that an agent intends to $\varphi$ if and only if there is an intention $J\in {\mathsf {Int}}$ such that *J* is represented by $\varphi$.^9^ To avoid trivial cases we require that there be at least one intention *J* in ${\mathsf {Int}}$, that is, ${\mathsf {Int}}\ne \emptyset$.

Second, intentions are “conduct-controlling pro-attitudes” (Bratman 1987, p. 16). Since our desires are mere *potential* influencers of action, this separates our intentions from desires. If I have the intention of realizing a certain future state of affairs, and nothing interferes, I will try to achieve it. This means that our intentions involve a characteristic kind of *unconditional* commitment.^10^ We discuss this characteristic feature informally in the next section, and we provide a formal specification in Sect. 5.

We would like to stress that our study excludes the temporal extension of agency that intentions seem to guarantee or facilitate. In this regard, our focus is quite different from what is often the focus of both philosophical^11^ and logical^12^ research on this topic. Since we concentrate on norms of rationality that apply to our intentions and the role intentions play in controlling our actions, this temporal aspect is not essential for our current purposes.^13^ What distinguishes our conceptual analysis from these other works is that the latter often *presuppose* the consistency requirement that we are currently rectifying.

A quick note: formal philosophers, especially philosophical logicians, may be familiar with our models and wonder why no syntactical counterpart has been given. A logical language could certainly be provided to express the notions of agency, possibility, and intentionality. This could be done in the standard way, after which our findings could be stated in terms of validities in this logical language. However, since this is not essential for our *conceptual* analysis of the consistency requirements, it is best to leave such an enterprise for another occasion.

We now turn to norms of rationality that apply to our intentions by focusing on the demand for mutually consistent intentions. To get at the core of Bratman’s consistency requirement, the following passage is insightful: he writes that the consistency requirement on intentions

is the requirement that one’s overall set of intentions be consistent $\ldots$ Note that this demand for consistency is not just that each intention have a consistent content; it includes, as well, the demand that one be able to agglomerate one’s various intentions into an overall intention that has a consistent content. (Bratman 2009b, p. 16)

To provide an accurate analysis of the consistency requirement, and to clarify the above passage, we put forward four formal consistency requirements, which correspond to different readings of this consistency requirement:

### **Definition 2**

*(Consistency Requirements)* Let $\langle W,{\mathsf {Act}}\rangle$ be a STIT model. Given a body of intentions ${\mathsf {Int}}$, we say that

(IC) the agent’s body of intentions is *internally consistent* if and only if every intention $J\in {\mathsf {Int}}$ is realizable, that is, $J\ne \emptyset$;
(MC) the agent’s body of intentions is *mutually consistent* if and only if any two intentions $J_1,J_2\in {\mathsf {Int}}$ are co-realizable, that is, $J_1\cap J_2\ne \emptyset$;
(OC) the agent’s body of intentions is *overall consistent* if and only if all intentions are jointly co-realizable, that is, $\bigcap _{J\in {\mathsf {Int}}} J\ne \emptyset$; and
(Agg) the agent’s body of intentions is *agglomerative* if and only if it is closed under intersections, that is, $\bigcap _{J\in {\mathsf {Int}}} J$ is an element of ${\mathsf {Int}}$.^14^

It is uncontroversial to consider having internally inconsistent intentions, at least knowingly, a case of irrationality.^15^ So we accept the norm of rationality which states that one’s intentions must be internally consistent. Given this internal consistency norm, we can see that these formal consistency requirements increase in strength: mutual consistency is the weakest requirement; agglomeration is the strongest requirement.^16^

### **Observation 1**

*Let*$\langle W,{\mathsf {Act}}\rangle$*be a STIT model*. *Let*${\mathsf {Int}}$*representthe agent’s body of intentions*. *Assume that*${\mathsf {Int}}$*is internally consistent.Then:*

*(OC) implies (MC), that is, if one’s body of intentions is overall consistent, then it is mutually consistent;*
*(Agg) implies (OC), that is, if one’s body of intentions is agglomerative, then it is overall consistent.*

## Successful operation and the acceptance criterion

Characteristic planning roles form the basis of Bratman’s functional analysis of our planning agency.^17^ To avoid complexities we restrict our attention to the fundamental *coordinating role*. It is useful to see how Bratman justifies norms of consistency by appealing to this coordinating role:

insofar as one’s intentions are inconsistent with each other and/or with one’s beliefs, this planning system will fail in its coordinating role, a role that is at the heart of the cross-temporal effectiveness of that system. So, in general, conformity to norms of consistency and means-end rationality are conditions for successful operation of this system of coordinated control. (Bratman 2009b, p. 17)

Bratman’s argument for accepting the consistency requirement then runs as follows: (1) conformity to the consistency requirement is a condition for successful operation; (2) because of the importance of successful operation and because of (1), the consistency requirement should be accepted. The argument in (2) is the basis of our acceptance criterion. Pre-formally, this argument says that for any norm that applies to an agent’s body of intentions, if conformity to this norm guarantees *successful operation*, then, because of the importance of successful operation, this norm should be accepted. Let us discuss this acceptance criterion in more detail.

What does the successful operation of our planning system consist in? If an agent intends to get some broccoli, it could be rational for her to go to a grocery store. If, in addition, she intends to call her dad, this could rationally impose the commitment to bring a mobile phone so she can call him on her way to the grocery store. It is important to note that, although these intentions may not be realized, the agent is still able to act in a way that is faithful to the commitments that rationally derive from each intention.

Conversely, suppose an agent intends to get some broccoli and she intends to get some cauliflower, but she only has a dollar in her pocket. Furthermore, suppose that upon arriving at the grocery store the agent finds out that these vegetables are jointly too expensive.^18^ This is a practical problem: in light of her intention to get some broccoli, the agent should spend her dollar on buying the broccoli, likewise for the cauliflower. Hence, she cannot act in a way that is faithful to each intention.^19^

We are not concerned with whether an agent’s body of intentions guarantees that she will *actually* choose an action whereby she promotes successful operation. Rather, we view the consistency requirements as norms regarding the coherence of the body of intentions. An agent’s body of intentions is required to *facilitate* successful operation. In a given scenario, this means that an agent’s body of intentions is sufficiently coherent for her to be able to promote successful operation. Each of her intentions induces a commitment to perform an action that is faithful with respect to it, so her body of intentions needs to be such that she is able to act in a way that is faithful to each of these commitments.

### **Definition 3**

*(Facilitating Successful Operation)* A given agent’s body of intentions *facilitates successful operation* if and only if there is a way for her to act faithfully with regard to each of her intentions. Conversely, her body of intentions does not facilitate successful operation if and only if no available action survives the filtering by all of her intentions.^20^

The pre-formal acceptance criterion for norms that apply to an agent’s body of intentions may now be derived from this conception of successful operation. We submit that such a norm is to be accepted if and only if conforming to it entails that one is able to promote successful operation. For example, the mutual consistency requirement is to be accepted if and only if whenever an agent’s body of intentions is mutually consistent she is able to act in a way that is faithful to each of her intentions.

### **Definition 4**

*(Acceptance Criterion)* A norm that applies to an agent’s body of intentions is *acceptable* if and only if in all possible scenarios where an agent’s body of intentions conforms to that norm it facilitates successful operation – that is, if and only if in all such cases there is an action available that survives the filtering by all of her intentions.

## Commitment, optimality, and practical consistency

To formally characterize the rational commitments that result from the adoption of a given intention, we propose using concepts from the theory of decision making under uncertainty.^21^ In particular, we propose adopting a dominance principle, which incorporates the “sure-thing” principle. Leonard Savage, who coined this principle, writes:

I know of no other extralogical principle governing decisions that finds such ready acceptance. (Savage 1972, p. 21)^22^

We could have adopted other decision-theoretical principles to argue for our central claims, but the “sure-thing” principle has the benefit of simplicity and acceptability. (In Sect. 6.3 we reflect on whether our results can be transferred to other decision principles.) The proposed dominance ordering concerns the realization of an intention rather than the realization of payoffs, as is standard in decision theory. To illustrate the sure-thing principle and the subsequent formal definitions, we use a running example.

**Fig. 1:**
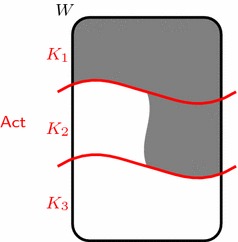
The peculiar gamble

Imagine a situation where an agent is faced with three options at a particular moment: taking 5 dollars, leaving with nothing, or gambling. If she chooses to gamble, we suppose that there is a possible world in which she gets 5 dollars and another possible world in which she gets nothing. Suppose the agent intends to get 5 dollars. This peculiar gambling situation is depicted in Fig. 1; what makes this case peculiar is that the agent can guarantee that her intention will be realized by declining the gamble.^23^ Here, $K_1$ represents the option of getting 5 dollars, $K_2$ the option of gambling, and $K_3$ the option of leaving. The grey area represents her intention, which is characterized by the possible worlds in which she gets 5 dollars. It should be clear that the agent can guarantee that her intention will be realized, namely by choosing $K_1$.

It should be clear that choosing to take the money is the most preferred option: why would the agent risk failing to realize her intention if she can *guarantee* that it will be realized by taking the money?^24^ From an intuitive point of view, the agent would be irrational if she intended to get 5 dollars yet chose to gamble or leave. Notice also that choosing to gamble is preferable to leaving: if the agent has the *chance* to get 5 dollars, why would she guarantee that her intention will not be realized by choosing to leave?

This brief discussion justifies the idea that an action *Kweakly dominates*$K'$ with respect to the intention *J* (notation: $K\preceq _J K'$) if and only if *K* promotes the realization of *J* at least as well as $K'$. That is, *K* weakly dominates $K'$ with respect to *J* if and only if, when *K* might result in realizing *J*, $K'$ guarantees that *J* will be realized—in other words, if and only if $K'$ surely results in an outcome that is at least as good as any outcome *K* may lead to. This constitutes an ordering on the available actions. The *optimal actions* are defined in terms of this ordering: an action *K* is optimal with respect to *J* if and only if there is no other action available that better promotes the realization of *J*.

### **Definition 5**

*(Dominance and Optimality)* Let $\langle W,{\mathsf {Act}}\rangle$ be a STIT model. Given actions *K* and $K'$, and an intention *J*, we say that

- *Kis weakly dominated by*$K'$*with respect toJ*, notation $K\preceq _J K'$, if and only if $K\cap J\ne \emptyset$ implies that $K'\subseteq J$;
- *Kis dominated by*$K'$*with respect toJ*, notation $K\prec _J K'$, if and only if $K\preceq _J K'$ and $K\nsucceq _J K'$; and
- *Kis optimal with respect toJ* if and only if *K* is not dominated with respect to *J*, that is, there is no $K'\in {\mathsf {Act}}$ such that $K\prec _J K'$.^25^

Because the dominance ordering concerns the realization of an intention rather than payoffs, the available actions naturally fall apart in three categories: actions that guarantee that the intention will be realized; actions that do not guarantee but are compatible with the realization of the intention; and actions that are incompatible with realizing the intention:

### **Observation 2**

(Tripartitioning Induced by Dominance) *Let*$\langle W,{\mathsf {Act}}\rangle$*be a STIT model. With respect to an intention toJ, the dominance ordering induces a partitioning of the available actions into (at most) three classes:*

- *actions that guarantee the realization ofJ, that is,actionsKthat satisfy*$K\subseteq J$;
- *actions that are compatible with realizing* *J* *yet fail to guarantee it, that is, actions* *K* *that satisfy* $K\cap J\ne \emptyset$ *and* $K\nsubseteq J$ *; and*
- *actions that are incompatible with realizingJ, that is, actionsKthat satisfy*$K\cap J=\emptyset$.^26^

Although Observation 2 does not mark a deep technical result, it gives us a neat characterization of the rational commitments that result from the adoption of a given intention. Suppose an agent intends to realize *J*. We submit that this intention imposes an *unconditional commitment* to perform an action that is optimal with respect to *J*. It may be helpful to consider two cases. First, if the agent is able to guarantee that *J* will be realized, she is required to perform an action that guarantees its realization. Second, if the agent is unable to guarantee that *J* will be realized, she is required to perform an action that is compatible with realizing *J*.^27^

### Practical consistency

An agent may not be able to *guarantee* successful operation. That is, there may not be an action available to her that guarantees the realization of each of her intentions. Still, some of her actions *promote* successful operation more than others. For example, an agent who intends to $\varphi$ while performing an action that is not optimal with respect to $\varphi$ does not promote successful operation. Therefore, we submit that an agent fails to promote successful operation if and only if she chooses an action that is not optimal with respect to one of her intentions. Or, conversely, an agent *promotes successful operation* if and only if she chooses an action that is optimal with respect to each of her intentions. What does this imply for norms that apply to the body of intentions? If an agent is to be able to be unconditionally committed to the realization of each of her intentions, her body of intentions must be such that she can act in a way that is optimal with respect to each of her intentions.

#### **Definition 6**

*(Practical Consistency)* Let $\langle W,{\mathsf {Act}}\rangle$ be a STIT model. Given a body of intentions ${\mathsf {Int}}$, we say that

(PC) the agent’s body of intentions is *practically consistent* if and only if there is an action *K* such that for every $J\in {\mathsf {Int}}$ it holds that *K* is optimal with respect to *J*.

## Analyzing norms of rationality

### Rejecting the consistency requirements

Our first central result shows that conforming to all three consistency requirements is insufficient for successful operation. Hence, these three consistency requirements should be rejected. Moreover, our second central result asserts that conformity to the practical consistency requirement is both sufficient and necessary for our planning system to fulfil its coordinating role.

**Fig. 2:**
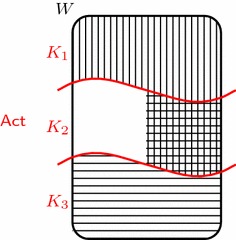
The grocery store: buy the broccoli, buy the cauliflower, or take a gamble on winning both?

To show this, let us amend the story of the agent (let us call her Ann). Suppose that Ann enters a grocery store with only one dollar in her pocket. She faces a dilemma: she only has one dollar to spend, but the broccoli and the cauliflower jointly cost more than one dollar. Suppose the shop owner recognizes Ann’s predicament and decides to offer her the option of gambling. If she gambles, we assume she has to pay the dollar and that there is a possible world in which she gets both vegetables but also another possible world in which she loses and gets nothing. Furthermore, suppose Ann’s body of intentions consists of three intentions: one, say $J_1$, to get broccoli, another, say $J_3$, to get cauliflower, and lastly, say $J_2$, to get both vegetables. The situation can thus be depicted as in Fig. 2. Here, $K_1$ through $K_3$ represent the options of only buying the broccoli, of accepting the gamble, and of only buying the cauliflower, respectively. The area filled with vertical lines represents Ann’s getting the broccoli, the area filled with horizontal lines represents her getting the cauliflower, and their intersection represents her getting both vegetables.

Is Ann’s body of intentions sufficiently coherent? Note that she conforms to all three formal consistency requirements because her intentions are agglomerative, that is, $J_1\cap J_2\cap J_3=J_2\ne \emptyset$. Now, let us use Observation 2 to examine the rational commitments that derive from the corresponding intentions: with respect to intention $J_1$, only action $K_1$ is optimal. Unfortunately, only action $K_2$ is optimal with respect to $J_2$. To make matters even worse, only action $K_3$ is optimal with respect to $J_3$. Hence, no action survives the filtering by her intentions. So, despite conforming to the consistency requirements, she faces an insurmountable dilemma: her planning system fails in its coordinating role. This shows that the consistency requirements are *insufficient* for facilitating successful operation.

#### **Result 1**

*All three consistency requirements are insufficient for facilitating successful operation. In light of the acceptance criterion, they should therefore be rejected.*

We can foresee two possible objections. First, one may object that the chosen dilemma is not genuine: one may be convinced that an agent cannot intend to win a gamble. In reply to this critique, we point out that this particular example is merely an instance of a collection of structurally similar cases, that is, those that share the structure illustrated in Fig. 2. The key is that it is a scenario of choice under *uncertainty*, so the objector has to argue that *any* such example is dubious.

To illustrate the difficulty of this task, the example can be amended to show that mutual consistency should be rejected even if the realization of each of the agent’s intentions is within her control. Note that (1) the agent is able to guarantee that intention $J_{1}$ will be realized, and likewise for intention $J_{3}$, by performing action $K_{1}$ and $K_{3}$, respectively; and (2) intentions $J_{1}$ and $J_{3}$ are co-realizable, since $J_1\cap J_3\ne \emptyset$. Let us denote $\{J_{1},J_{3}\}$ by ${\mathsf {Int}}^{*}$. We immediately see that ${\mathsf {Int}}^{*}$ is mutually consistent and that the agent is able to guarantee the realization of each intention separately. Since we already saw that only $K_1$ is optimal with respect to $J_1$ and only $K_3$ is optimal with respect to $J_3$, ${\mathsf {Int}}^{*}$ unfortunately does not facilitate successful operation. The claim that the mutual consistency requirement is to be rejected is therefore sustained even in cases where the attainment of each of the intentions separately is within the agent’s control.

Second, one may object that we need not be so reckless in rejecting the three consistency requirements, for they can be strengthened to facilitate successful operation. This objection invites two replies. First, even if the consistency requirements can be appropriately extended, this hardly seems to justify their acceptance. It seems that anything goes once we start endorsing norms that could be sufficiently strengthened. Second, according to this objection, our argument is interpreted as showing that the consistency requirements are *too weak*. In the next subsection, however, it is argued that conformity to the consistency requirements is also unnecessary when it comes to the ability of our intentions to facilitate successful operation. This means that any acceptable extension will be *too strong*. Our results thus entail that the consistency requirements are neither too weak nor too strong; instead, they miss their mark on a more fundamental count: they fail to enable the successful guidance of our actions.

Does the norm of practical consistency fare any better in this example? Since Ann’s body of intentions does not conform to the practical consistency requirement, the answer is affirmative. In general, if we assume that the technical notion of optimality characterizes the unconditional commitment distinctive of intentions, then we can assert that the practical consistency requirement is both sufficient and necessary for facilitating successful operation:

#### **Result 2**

*The norm of practical consistency is both sufficient and necessary for facilitating successful operation. Therefore, in particular, the norm of practical consistency is to be accepted.* 28

Although this result is a formal triviality, it marks conceptual progress. The sufficiency claim shows that, whereas traditional consistency requirements fail to support the central planning roles, our practical consistency requirement succeeds in doing so. Furthermore, the necessity claim shows that, insofar as one’s intentions are practically inconsistent, one’s planning system will fail in its coordinating role. So, in general, conformity to the practical consistency requirement is a condition for successful operation.

### Rational mutually inconsistent intentions

Our third central result demonstrates that an agent’s planning system can get along perfectly despite violating the mutual consistency requirement. Hence, conformity to any consistency requirement is unnecessary for a successful planning system, and it can therefore be rational to have mutually inconsistent intentions.

**Fig. 3:**
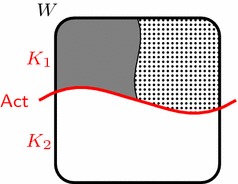
The cookie store: violating the mutual consistency requirement need not break down one’s planning system

To show this, let us imagine that Britney enters a cookie store, where she is faced with two options: to gamble or not to gamble. If she chooses to gamble, we assume that she is guaranteed to win exactly one of two prizes: a doughnut or a cookie. In particular, if she chooses to gamble, we suppose that there is a possible world in which she gets a doughnut and another possible world in which she gets a cookie. If she chooses not to gamble, we suppose she has no chance of winning any of these prizes. Furthermore, we suppose that Britney’s body of intentions consists of two intentions: one, say $J_1$, to get a doughnut, and the other, say $J_2$, to get a cookie.^29^ This scenario can thus be presented as in Fig. 3. Here, $K_1$ and $K_2$ represent the options of gambling and not gambling, the grey area represents her getting a doughnut, and the dotted area represents her getting a cookie.

It should be clear that Britney’s body of intentions is not mutually consistent. Since mutual consistency is the weakest of the three alternative formalizations of the consistency requirement (Observation 1), her body of intentions also fails to be overall consistent and fails to be agglomerative. However, in light of Observation 2 it is easy to see that gambling is optimal with respect to each of her intentions. So although her intentions are mutually inconsistent, they do not pose a practical dilemma: both recommend gambling. Hence, her intentions facilitate successful operation and thus provide the characteristic coordinating role. We conclude that she is rational despite having mutually inconsistent intentions.

#### **Result 3**

*None of the three consistency requirements is necessary for facilitating successful operation. Therefore, it can be rational to have mutually consistent intentions.*

Together with Result 1, this shows that the consistency requirements are neither too weak nor too strong. Our new practical consistency requirement adequately corrects this failure: conformity to it is both sufficient and necessary for an effective planning system. We contend that the consistency requirements are the wrong type of norm: whereas they focus on rendering the *contents* of the intentions consistent, the practical consistency requirement demands that our intentions be able to simultaneously and unconditionally *guide* our action.

### A brief reflection on the adopted dominance ordering

Let us briefly reflect on the adopted dominance ordering (see Definition 5 and Observation 2). One may ask whether our results are preserved if the characteristic commitment of an agent’s intentions is modelled by adopting a different decision principle (for example, maximizing expected utility). It is useful to point out that Results 1 and 3, which state that all three consistency requirements are neither sufficient nor necessary for a successful planning system, only depend on the following two properties of the adopted decision principle:

1. When an agent intends to realize *J* and she is able to guarantee that *J* will be realized, then the decision principle requires her to guarantee that *J* will be realized;
2. When an agent intends to realize *J* and there is exactly one action available to her that is compatible with realizing *J*, say *K*, then the decision principle requires her to perform *K*.^30^

It can easily be shown that our discussion of Fig. 3 only uses the second of these properties to argue that all three consistency requirements are unnecessary, that is, Result 3. Moreover, in our treatment of the first of two anticipated objections, we have argued that the mutual consistency requirement should be rejected even in cases where the attainment of each of the intentions is within the agent’s control. This implies that only the first property is needed to argue that all three consistency requirements are insufficient, that is, Result 1.

The case for Result 2, which states that the practical consistency requirement is both sufficient and necessary, is more subtle. Since our practical consistency requirement refers to the adopted dominance ordering, say $D_1$, it is unlikely that this result is preserved when a different decision principle, say $D_2$, is adopted to model the characteristic commitment of our intentions.^31^ That is, there may be scenarios in which one’s intentions are practically consistent, yet where no action survives the filtering by all intentions using decision principle $D_2$. Although this suggests that the practical consistency requirement is intimately linked to the adopted dominance ordering $D_1$, it means that the practical consistency requirement can be straightforwardly amended using one’s preferred decision principle $D_2$: an agent’s intentions are $D_2$-practically consistent if and only if there is an action available that is $D_2$-optimal with respect to each of her intentions. For example, for expected utility theory (EU) this translates to the following: an agent’s intentions are EU-practically consistent if and only if there is an action available that maximizes the expected utility with respect to each of her intentions.^32^

What this brief reflection shows, then, is that anyone who subscribes to the first and second properties needs to amend the consistency requirement. Instead, her body of intentions should be $*$-practically consistent.^33^ This reinforces the central claim that the consistency requirements need to be rectified.

## The own-action condition

Do our results rely on scenarios of choice under *uncertainty*? To answer this question, we investigate a particular subclass of intentions. In theorizing about intentions, it is natural to distinguish between intentions that violate and intentions that comply with the ‘own-action condition’. This condition incorporates the intuition that an agent can only intend her own actions, not contingencies.^34^ Michael Bratman writes that

according to this own-action condition it is always true that the *subject* of an intention is the *intended agent* of the intended activity. (Bratman 2014, p. 13)^35^

In taking up his suggestion, we propose that the content of an intention that conforms to the own-action condition is composed of actions of the agent herself. The content of such an intention is that the agent herself sees to it that $\varphi$ holds. On this view, when Ann intends that she closes the door, the content of her intention is that she constrains the possible worlds to those where the door is closed. A world in which Bob closes the door, for instance, fails to realize her intention. Hence, the intention is realized if and only if a closing-the-door action is performed by Ann herself. So the content is specified by the union of closing-the-door actions.

### **Definition 7**

*(Own-Action Condition)* Let $\langle W,{\mathsf {Act}}\rangle$ be a STIT model. Let *J* be an intention. We say that

(OAC) *Jconforms to the own-action condition* if and only if there is a subset of actions ${\mathsf {Act}}'\subseteq {\mathsf {Act}}$ such that *J* is realized if and only if one of the actions in ${\mathsf {Act}}'$ is performed, that is, $J=\bigcup _{K\in {\mathsf {Act}}'} K$.^36^

An interesting result emerges when our new analysis is restricted to intentions that conform to the own-action condition:

### **Result 4**

*If an agent’s intentions are internally consistent and conform to the own-action condition, then the overall consistency requirement and the practical consistency requirement are equivalent.*

*That is, for any STIT model* $\langle W,{\mathsf {Act}}\rangle$ *and any body of internally consistent intentions* ${\mathsf {Int}}$ *that conform to the own-action condition, the following are equivalent:*

(OC) $$\bigcap _{J\in {\mathsf {Int}}} J\ne \emptyset$$ *, and*
(PC) *there is an actionKsuch that for every*$J\in {\mathsf {Int}}$*it holds thatKis optimal with respect toJ*.

Combined with Result 2, this implies that when we restrict our analysis to intentions that conform to the own-action condition, the overall consistency requirement is to be accepted. Hence, our new analysis shows that for such intentions, conformity to the overall consistency requirement is a condition for successful operation. So, in particular, such rational intentions are mutually consistent.

This highlights that the consistency requirements only need to be rectified for intentions that violate the own-action condition. To investigate such intentions and the norms of rationality that govern them, our new conceptual analysis relies on choice under uncertainty, as studied in decision theory. So in scenarios of choice under uncertainty, Bratman’s consistency requirements need to be amended to guarantee an effective planning system.

### General intentions

One might have reason to think that intentions need to conform to the “own-action condition”. Michael Bratman, however, envisions a planning theory of intentions, and of norms that apply to our intentions, that encompasses intentions that violate the own-action condition. To see this, note that his analysis of cases of modest sociality crucially relies on the condition that we each “intend that we act” (Bratman 2014).^37^ He acknowledges that such intentions violate the own-action condition.^38^ However, in attempting to retain continuity between analysing our individual planning agency and cases of modest sociality, he dismisses the idea that “intending that we act” and “intending to act” are two fundamentally different attitudes:

The distinction is not between two fundamentally different attitudes, but between two different kinds of *contents* of the attitude of intending, an attitude described by the planning theory. ...Both *intending to act* and *intending that we act* play plan-theoretic roles and are subject to associated norms of plan rationality. (Bratman 2014, p. 14 – emphasis added)

Bratman hence proposes that “intending that we act” and “intending to act” are fundamentally the same; they only differ in terms of their content. Among other things, the former violates the own-action condition, while the latter respects it. Since he argues that both are subject to associated norms of plan rationality, his consistency requirement is meant to also apply to intentions that violate the own-action condition.

Our conceptual analysis, however, showed that although intentions that conform to the own-action condition are subject to the overall consistency requirement, intentions in general need not be, thereby rebutting his claim that both “intending that we act” and “intending to act” are subject to his consistency requirement. To finish on a positive note, we offer a way out of his predicament: he should adopt the new practical consistency requirement. Intentions need to conform to the practical consistency requirement, regardless of their content.

## Discussion

We have rebutted the idea that an agent is irrational if her intentions are not consistent. We have traced the justification for the consistency requirement to a deeper norm of successful operation. Given the norm of successful operation, however, it has been shown that the consistency requirement should be rectified. In particular, there are cases in which successful operation is supported even though the agent’s intentions are mutually inconsistent. We have therefore defended the idea that we should endorse the norm of practical consistency, which demands that one’s intentions be able to simultaneously and unconditionally guide one’s action. Finally, under the own-action condition, we have seen that the new practical consistency requirement coincides with the traditional consistency requirement.

## Appendix: Proofs

### Observation 2

(Tripartitioning Induced by Dominance) *Let*$\langle W,{\mathsf {Act}}\rangle$*be a STIT model*. *With respect to an intention to**J**, the dominance ordering induces a partitioning of the available actions into (at most) three classes:*

- *actions that guarantee the realization of* *J* *, that is,* *actions* *K* *that satisfy* $K\subseteq J;$
- *actions that are compatible with realizing* *J* *yet fail to guarantee it, that is, actions* *K* *that satisfy* $K\cap J\ne \emptyset$ *and* $K\nsubseteq J$ *; and*
- *actions that are incompatible with realizing* *J* *, that is,* *actions* *K* *that satisfy* $K\cap J=\emptyset .$

### *Proof*

First, note that these classes constitute a partitioning. Second, note that, depending on *J*, some of these classes might be empty. Finally, we show that the first dominates the second, and the second dominates the third:

Take an arbitrary intention *J*. Let $K_1$ through $K_3$ be actions such that $K_1\subseteq J$, $K_2\cap J\ne \emptyset$ and $K_2\nsubseteq J$, and $K_3\cap J=\emptyset$. Then:

1. $K_1\succ _J K_2$: $K_1\succeq _J K_2$ follows from the fact that $K_1\subseteq J$. The converse does not hold, since $K_1\cap J=K_1\ne \emptyset$ yet $K_2\nsubseteq J$.
2. $K_2\succ _J K_3$: $K_2\succeq _J K_3$ follows from the fact that $K_3\cap J=\emptyset$. The converse does not hold, since $K_2\cap J\ne \emptyset$ yet $K_3\nsubseteq J$.

$$\square$$

### **Result 4**

*If an agent’s intentions are internally consistent and conform to the own-action condition, then the overall consistency requirement and the practical consistency requirement are equivalent.*

T*hat is, for any STIT model *$\langle W,{\mathsf {Act}}\rangle$*and any body of internally consistent intentions *${\mathsf {Int}}$*that conform to the own-action condition, the following are equivalent:*

(OC) $$\bigcap _{J\in {\mathsf {Int}}} J\ne \emptyset$$ *, and*
(PC) *there is an action* *K* *such that for every* $J\in {\mathsf {Int}}$ *it holds that* *K* *is optimal with respect to* *J.*

### Proof

It follows immediately from the definition of the own-action condition and Observation 2 that when an intention *J* conforms to the own-action condition, then an action *K* is optimal with respect to *J* if and only if $K\subseteq J$.

$\Downarrow$: Assume (OC). Take an arbitrary $w\in \bigcap _{J\in {\mathsf {Int}}} J$. Since ${\mathsf {Act}}$ constitutes a partitioning of *W*, there is but one $K\in {\mathsf {Act}}$ such that $w\in K$; denote this action by $K_w$. For any $J\in {\mathsf {Int}}$, let ${\mathsf {Act}}_J\subseteq {\mathsf {Act}}$ be such that $J=\bigcup _{K\in {\mathsf {Act}}_{J}} K$. Since $w\in J=\bigcup _{K\in {\mathsf {Act}}_{J}} K$ for any $J\in {\mathsf {Int}}$, this implies that $K_w\subseteq J$ for any $J\in {\mathsf {Int}}$. Hence, for every $J\in {\mathsf {Int}}$ it holds that $K_w$ is optimal with respect to *J*. So (PC) holds.
$\Uparrow$: Assume (PC). Let *K* be an action such that for every $J\in {\mathsf {Int}}$ it holds that *K* is optimal with respect to *J*. Then it holds that $K\subseteq J$ for every $J\in {\mathsf {Int}}$. Hence, $\bigcap _{J\in {\mathsf {Int}}} J\supseteq K\ne \emptyset$. So (OC) holds.

$$\square$$

## Footnotes

1 On several occasions, Bratman (1987, 2009a, b, 2014) has argued that norms apply to rational intentions.

2 Some philosophers have also argued in favour of our main claim. Our argument is most closely related to McCann’s (1991). Whereas we focus on Bratman’s arguments in favour of the consistency requirements, McCann’s arguments focus on the similarities between settled objectives and intentions with regard to the functional role of intentions in deliberation and action.

3 It is important to note that weighing an agent’s various intentions is not an option if each of her intentions is to induce an *unconditional* commitment. Aggregation procedures thus cannot resolve the conceptual issue at hand.

4 STIT theorists are ambiguous about whether we ought to interpret ${\mathsf {Act}}$ in terms of choices or actions. According to Horty (1996, p. 274—emphasis added), ${\mathsf {Act}}$ “is a device for representing the constraints that an agent is able to exercise upon the course of history at a given moment, the *actions or choices* open to him at that moment”. Belnap et al. (2001, pp. 33–34—notation adapted and emphasis added) writes: “The equivalence classes belonging to ${\mathsf {Act}}$ can be thought of as the possible *choices or actions* available.” For our current purposes, the elements of ${\mathsf {Act}}$ are best thought of as actions.

5 For example, Perloff and Belnap (2011, pp. 583–584) write: “Part of the idea of indeterminism as we conceive it is that at any given moment there are a variety of ways in which the world might proceed. Such possibilities are real, not merely epistemic; they are possibilities *in re*.”

6 These future-directed intentions (such as my intention to submit this paper by the end of the month) have been distinguished from intentions in action (such as my typing with the intention of finishing this introduction) and intentional acts (such as my typing these words intentionally) (see Anscombe 1963).

7 It is important to flag that not all characteristics of future-directed intentions are essential for our purposes. For instance, the characteristic temporal stability of future-directed intentions is inessential for us.

8 Tuomela (2005, pp. 329–330) calls for a similar distinction between “action intentions” and “aim intentions”.

9 It is important to note that we employ *neighbourhood* semantics, which reads as ‘*J* is represented by $\varphi$’. Technically, this means that $J=\{w\in W\mid w$ is a $\varphi$-world$\}$ (note the equality sign). If we had employed the standard possible worlds semantics, it would have read ‘*J* consists only of $\varphi$-worlds’, meaning $J\subseteq \{w\in W\mid w$ is a $\varphi$-world$\}$. Neighbourhood semantics have been introduced in the modal logic community independently by Montague (1970) and Scott (1970); Chellas (1980) provides a textbook treatment of the subject. Konolige and Pollack (1993) were the first to model intentions using neighbourhood semantics instead of the standard possible worlds semantics. Footnotes 14 and 26 provide our main reasons for preferring neighbourhood semantics over standard possible world semantics.

10 According to Bratman (1987, Chapter 5) intentions can occasionally be revised. Intention revision is one of the central themes of Bratman, Israel, and Pollack (1988, especially Sections 4 and 5). However, note that Bratman (1987, pp. 16–17) writes: “My intention resists reconsideration: it has a characteristic *stability* or *inertia*. ...Retention of my prior intention and nonreconsideration is, so to speak, the ‘default option.’”

11 Bratman (2009b, p. 26) writes: “Planning agency is a distinctive form of agency, one that contributes substantially to the pursuit of complex, temporally extended aims, to structures of self-governance, and to forms of sociality.”

12 See, for instance, the work of Cohen and Levesque (1990) and Meyer et al. (1999).

13 In this respect, the presuppositions of our formal analysis are roughly similar to Van Hees and Roy (2008, p. 1), who “attempt to model the role of intentions in decision making”.

14 Items (IC) and (Agg) resemble the formal notions in Axioms 1 and 2 of Van Hees and Roy (2008, pp. 210–211): formalizations of “feasibility” and “agglomerative”, respectively. Note, however, that we do not wish to impose an analogue of their third axiom of “intention logical omniscience”.

15 Note that $J=\emptyset$ if and only if *J* is represented by the formula $\bot$ (absurdity). This means that having internally inconsistent intentions amounts to intending the absurd.

16 Only non-trivial, omitted proofs of observations and results are given in the “Appendix”, that is, only the proofs of Observation 2 and Result 4. Since the proof of the following observation is rather trivial, we decided to exclude it.

17 For instance, Bratman (2009a, p. 411) writes: “The planning theory of intention and of our agency highlights the fundamental coordinating and organizing roles of structures of planning in the temporally extended and social practical thought and action of agents like us.”

18 It is straightforward to alter the story in such a way that it concerns not epistemic uncertainty, but ontological uncertainty. Our argument can thus be construed both epistemically, relating beliefs to intentions, and ontologically, relating possibilities to intentions.

19 Our study does not concern revising one’s intentions; instead, we view the norms that apply to our intentions as a coherence condition on one’s body of intentions. This is in line with Nozick’s (1993, p. 140) supposition about norms concerning the structure of our preferences: “although [contemporary decision theory] does not say that any individual preference is irrational, it does say that a group of them together can be. Let us suppose that there are normative principles specifying the structure of several preferences together and that these principles are conditions of rationality.”

20 One might consider resolving the competition between an agent’s various intentions by weighing or aggregating them. This would, however, be at odds with the characteristic unconditional commitment of her intentions. This unconditional commitment entails that none of her intentions can be compromised. Indeed, to facilitate successful operation there has to be a way to act faithfully with regard to every one of her intentions – without concessions.

21 Our personal inspiration comes from the field of deontic logic, where Horty (1996) has proposed a similar analysis for representing what an agent ought to do. We shall not engage in an in-depth comparison of the current approach to intentions and the approach in deontic logic because it would lead us too far astray.

22 In their axiomatic approach to decision theory, Luce and Raiffa (1957, see Section 13.3 and p. 306) express the dominance principle in Axiom 5 and write: “Axioms 1 through 5 seem quite innocuous and, so far as we are aware, all serious proposals for criteria satisfy them.”

23 The example is inspired by The Gambler’s Problem discussed by Horty (1996, p. 288).

24 Compare Roy (2009, p. 338): “I take ‘providing a filter of admissibility’ to mean ruling out options that are incompatible with the agents achieving their intentions.” Our proposal is more fine-grained since actions that guarantee the realization of an outcome in *J* are preferred over those that are merely compatible with *J*.

25 It can easily be shown that this explication of weak dominance with respect to an intention *J* corresponds to the standard decision-theoretic notion for single-agent decision problems with respect to the utility function $u:W\rightarrow \{0,1\}$ given by $u(w)=1$ if and only if $w\in J$ (see for instance Horty 2001, pp. 59–69).

26 It is interesting to note that an action *K* can be optimal with respect to *J* and yet fail to be optimal with respect to a superset $J'\supset J$. This is one of the reasons to reject an analogue of the third axiom of Van Hees and Roy (2008, p. 211), which corresponds to “intention logical omniscience”.

27 The adopted dominance ordering ignores quantitative aspects. In particular, if the agent is unable to guarantee the realization of her intention, then *every* action that is compatible with realizing her intention is optimal. For example, an action would be optimal even if there were another action available that had a higher probability of leading to the realization of her intention. It is important to note, however, that this is *not* essential to our results; see Sect. 6.3 and Property 2 in particular.

28 In Sect. 6.3 we show how to revise the practical consistency requirement when the unconditional commitment would be modelled using a different decision principle.

29 It has been suggested to the authors that instead of having two separate intentions, the agent in this scenario should have a single intention, namely $J_1\cup J_2$. Our brief reply is twofold. First, our analysis can be applied to show that an agent who intends only $J_1\cup J_2$ is indeed also rational. Nonetheless, our analysis points out that a body of intentions consisting only of $J_1$ and $J_2$ is already practically consistent. Secondly, we are investigating *norms* of rationality that apply to our intentions. Suppose this suggestion is generalized to a norm demanding that an agent intend that at least one of her intentions will be realized. The current analysis could then be applied to show that this norm is to be rejected (for instance, by extending the body of intentions in Fig. 2). Providing this analysis is, however, beyond the scope of this paper.

30 One may find the adopted dominance ordering unintuitive because it does not distinguish between any two actions that are compatible with realizing the intention (see Footnote 27). In this light, it is important to note that this second property only concerns cases where there is *exactly one* action available that is compatible with realizing the intention.

31 For example, Duijf (forthcoming, Section 2) adopts the decision-theoretic principle of *admissibility* to model the characteristic unconditional commitment akin to our intentions. Admissibility has a long tradition in decision theory [see the discussion by Kohlberg and Mertens (1986, Section 2.7)].

32 Broersen (2011, p. 792) uses a probabilistic STIT framework to provide an analysis of attempts: “An attempt for $\varphi$ is a choice most likely leading to $\varphi$ given [the agent’s] subjective probabilities about what other agents choose simultaneously.”

33 Where the details are spelled out using your preferred decision principle, as indicated above.

34 Compare Audi (1973): “To distinguish intending to bring about $\varphi$ by doing *A* from merely hoping to bring about $\varphi$ by doing *A*, we need to require that *x* at least believe his doing *A* will be a probable way to achieve $\varphi$.”

35 Compare Mele (1989, p. 20, altered notation), who introduces “intention$*$” to denote “whatever is left of *i*’s intention to $\varphi$ when we substitute for her belief that she probably will $\varphi$ a belief that she probably will not $\varphi$.” Similarly, Tuomela (2005, p. 329) distinguishes “action intentions” from “aim intentions”: “In the latter case it is not required that the agent believes that he with some likelihood can alone bring about or see to it that the action or its result event comes about.”

36 Note that an agent can be able to guarantee the realization of his intention *J* despite the fact that *J* does not conform to the own-action condition, but not vice versa. This is easy to see since, formally, the former only means that there is an action *K* that satisfies $K\subseteq J$.

37 Bratman is not the only philosopher to acknowledge the importance of intentions that violate the own-action condition for the analysis of collective agency. Tuomela (2005, p. 330) analyses “joint intentionality” by relying on “we-intentions”, which are a sort of “aim intention”: “It can technically be said that a joint intention consists of the participants’ we-intentions about the existence of which the participants have mutual belief.”

38 For example, Bratman (2014, p. 13) writes: “Here I just want to acknowledge that the view I will be developing does involve rejecting this own-action condition, since it appeals to intentions of each individual participant that *they* (the group) act.”
